# Knowledge and attitudes about obstructive sleep apnea among otorhinolaryngology trainee residents in Saudi Arabia: A survey-based cross-sectional study

**DOI:** 10.12688/mep.19245.1

**Published:** 2022-08-17

**Authors:** Abdulrahman Alsaif, Khalid Aldilaijan, Mai Almasoud, Arulanantham Zechariah Jebakumar

**Affiliations:** 1 Department of Otorhinolaryngolgoy, King Abdulaziz Hospital, Alahsa, 31982, Saudi Arabia; 2 Department of Otorhinolaryngology, King Fahd Military Medical Complex, Dhahran, 31932, Saudi Arabia; 3 Vice Deanship of Postgraduate Studies Research, Prince Sultan Military College of Health Sciences, Dhahran, 31932, Saudi Arabia

**Keywords:** obstructive sleep apnea, knowledge, attitude, trainee, residents

## Abstract

Background: Obstructive sleep apnea is a common disorder frequently encountered in otorhinolaryngology practice. This study was conducted to assess the knowledge and attitudes toward obstructive sleep apnea among otorhinolaryngology trainees in Saudi Arabia.

Methods: This was a survey-based cross-sectional study. An online questionnaire was sent in July 2020 via email and WhatsApp instant messaging to all otorhinolaryngology trainee residents in Saudi Arabia. We utilized the previously validated obstructive sleep apnea knowledge and attitudes questionnaire (OSAKA).

Results: 32.4% of all email recipients completed the questionnaire and met the inclusion criteria. 66.7% were males, thirty-two participants (53.3%) were at the junior level (R2–R3), and twenty-eight (46.7%) were at the senior level (R4–R5). The mean total knowledge score was 13.98/18. Senior residents had a higher mean total knowledge score than junior residents. Senior residents had a higher mean total attitude score than junior residents. Age, gender, residency program area, and years of previous otorhinolaryngology practice showed no significant differences in terms of knowledge levels and attitudes toward obstructive sleep apnea. Exposure to obstructive sleep apnea surgery and awareness of sleep disorders other than sleep apnea were found to be associated with an increased level of confidence in identifying patients at risk of obstructive sleep apnea and in the ability to manage them.

Conclusions: This study describes the current condition of obstructive sleep apnea knowledge and attitudes among otorhinolaryngology residents in Saudi Arabia. Addressing studied elements may improve training outcomes.

## Introduction

Obstructive sleep apnea (OSA) is a common disorder worldwide ^1^. Its prevalence in Saudi adults is estimated to be approximately 8.8% ^2^. Previous questionnaire-based studies in middle-aged Saudis showed that almost 3 out of 10 Saudi men and 4 out of 10 Saudi women are at risk of OSA ^3, 4^. Although it leads to serious complications ^5– 9^, OSA remains an underdiagnosed and undertreated disorder ^10, 11^.

The obstructive sleep apnea knowledge and attitudes (OSAKA) questionnaire is an assessment tool used to assess physicians’ knowledge and attitudes regarding the identification and management of patients with OSA ^12^. It was designed and validated in 2003. Since then, it has been used to assess the knowledge and attitude of medical students and physicians from different specialties in different countries ^13– 18^.

Sleep medicine is one of the core specialty topics in the Saudi board for otorhinolaryngology (ORL). The evaluation and treatment of OSA is part of the learning and training objectives of the Saudi Board of ORL (SB-ORL) ^19^. However, to the best of our knowledge, no previous studies have assessed the knowledge and attitudes toward OSA among ORL trainee residents in Saudi Arabia. Therefore, this study aimed to assess the knowledge of and attitudes toward OSA among ORL trainee residents in Saudi Arabia.

## Methods

### Study design

We conducted a survey-based cross-sectional study. We utilized a previously validated questionnaire called the “OSAKA questionnaire” ^12^. The OSAKA questionnaire is composed of 18 items that are used to assess one’s knowledge and 5 items to assess one’s attitudes around dealing with OSA. The online questionnaire was sent in the period between July and August 2020 via email and WhatsApp instant messaging to the included subjects.

### Participants

The survey was sent to the Saudi board’s ORL trainee residents who were in training during the study period. Participants’ emails and WhatsApp numbers were collected from the chief residents in each area of the Kingdom. We sent the survey to those in their second (R2) to final year of residency (R5) (n = 185). First-year residency trainees were not included, as they were rotating in preparatory rotations and had no previous exposure to ORL clinical training. The survey was conducted anonymously.

### Data collection

Along with the OSAKA questionnaire, sociodemographic data and data about previous exposure to sleep practices were also collected. The sociodemographic data included age, gender, level of training, years of ORL practice including residency, year of graduation from medical college, and residency program region (by province). Data reflecting previous exposure to sleep practices were also collected, such as the frequency of exposure to diagnosed OSA patients, patients susceptible to OSA, polysomnography data, obese patients, and surgery directed to treat OSA. We also collected data on trainees’ self-reported awareness and previous consideration of other sleep disorders during their clinical training (such as inadequate sleep hygiene, insomnia, narcolepsy, and periodic limb movement disorder) as well as awareness of other related disorders (such as overlap syndrome and obesity hypoventilation syndrome). We considered those who answered that they were “aware of other sleep disorders” and who gave examples of other sleep disorders as the aware group.

The knowledge section of the OSAKA questionnaire was assessed by calculating the total true responses for the knowledge section. The total knowledge score was calculated out of 18.

The attitude section of the OSAKA questionnaire consisted of five questions (two for importance and three for confidence). It was calculated out of 5 for each question (the responses “extremely important” and “strongly agree” were scored as scores of 5). The total attitude score was calculated using a maximum score of 25, reflecting the sum score of the five attitude elements.

For the confidence questions, we considered those who answered “agree” and “strongly agree” as the confident group in identifying OSA-susceptible patients and managing OSA patients.

Exposure to each of the aforementioned clinical exposure items was divided and classified as either no exposure or any number of exposure incidences.

### Data analysis

Data and variables were coded and entered in a designed data entry file using the Statistical Package for the Social Services (SPSS) program version 22 for Windows. Knowledge levels and attitudes were calculated as the mean scores using an independent sample t-test. They were also calculated as medians and interquartile ranges (IQR). Using ANOVA, comparisons were performed between different ages, genders, residency program areas, levels of training, and years of previous ORL practice. Statistical significance was set at *p* < 0.05.

### Ethical statement

This study was approved by the institutional review board (IRB) of King Fahad Military Medical Complex – Dhahran, Saudi Arabia (IRB number AFHER-IRB-2020-014). Participant consent was obtained by electronic methods.

## Results

The full dataset can be found under *Underlying data* ^20^. Sixty trainee residents (32.4% of all questionnaire recipients) completed the questionnaire and met the inclusion criteria. Forty respondents (66.7%) were males. The mean age of the participants was 28 years. Thirty-two participants (53.3%) were at the junior level (R2–R3), and twenty-eight participants (46.7%) were at the senior level (R4–R5). Most of the respondents (40%) were from the eastern providence and Bahrain programs ( Table 1).

**Table 1.** Socio-demographic data.

	N (%)
**Gender** Males Females	40 (66.7%) 20 (33.3%)
**Level of training** R2 R3 R4 R5	18 (30%) 14 (23.3%) 14 (23.3%) 14 (23.3%)
**Level of residency** Junior (R2 and R3) Senior (R4 and R5)	32 (53.3%) 28 (46.7%)
**Residency program region** Almadina Eastern province and Bahrain Jeddah Makkah and Taif Riyadh Southern province	3 (5%) 24 (40%) 5 (8.3%) 4 (6.7%) 6 (16.7%) 14 (23.3%)
**Age (mean ± SD)**	(28.75 ± 2.24)

The mean total knowledge score was 13.98 ± 2.4 out of 18 ( Table 2). The median total knowledge score was 14 (IQR 12–16). One junior and one senior trainee resident each scored 18 out of 18. Twenty residents (33.3%) had a total knowledge score ≥ 16. Thirty-seven participants (61.7%) had a total knowledge score ≥ 14. Senior residents had a higher mean total knowledge score compared to junior residents (14.96 ± 1.7 vs. 13.19 ± 2.5, *p* = 0.02). Question 5 of the knowledge section "OSA is associated with hypertension" and question 11 of the knowledge section "A craniofacial and oropharyngeal examination is useful in the assessment of patients with suspected OSA" had the highest scores (100%), while question 3 of the knowledge section "The estimated prevalence of OSA among adults is between 2 and 10%" had the lowest scores (50%).

**Table 2.** Responses on the knowledge segment of the OSAKA questionnaire.

OSAKA Knowledge questions (correct response)	n (%) of correct responses			
	All participants N=60	Juniors N=32	Seniors N=28	*P*-value *
Item 1. Women with OSA may present with fatigue alone. **(T)**	43 (71.7%)	21 (65.6%)	22 (78.6%)	0.27
Item 2. Uvulopalatopharyngoplasty is curative for the majority of patients with OSA. **(F)**	38 (63.3%)	18 (56.3%)	20 (71.4%)	0.22
Item 3. The estimated prevalence of OSA among adults is between 2 and 10%. **(T)**	30 (50%)	14 (43.8%)	16 (57.1%)	0.30
Item 4. The majority of patients with OSA snore. **(T)**	44 (73.3%)	26 (81.3%)	18 (64.3%)	0.14
Item 5. OSA is associated with hypertension. **(T)**	60 (100%)	32 (100%)	28 (100%)	-
Item 6. An overnight sleep study is the gold standard for diagnosing OSA. **(T)**	53 (88.3%)	28 (87.5%)	25 (89.3%)	0.83
Item 7. CPAP (continuous positive airway pressure) therapy may cause nasal congestion. **(T)**	38 (63.3%)	17 (53.1%)	21 (75.0%)	0.08
Item 8. Laser-assisted uvuloplasty is an appropriate treatment for severe OSA. **(F)**	40 (66.7%)	15 (46.9%)	25 (89.3%)	0.001
Item 9. The loss of upper airway muscle tone during sleep contributes to OSA. **(T)**	53 (88.3%)	28 (87.5%)	25 (89.3%)	0.83
Item 10. The most common cause of OSA in children is the presence of large tonsils and adenoids. **(T)**	59 (98.3%)	32 (100%)	27 (96.4%)	0.28
Item 11. A craniofacial and oropharyngeal examination is useful in the assessment of patients with suspected OSA. **(T)**	60 (100%)	32 (100%)	28 (100%)	-
Item 12. Alcohol at bedtime improves OSA. **(F)**	39 (65.0%)	19 (59.4%)	20 (71.4%)	0.33
Item 13. Untreated OSA is associated with a higher incidence of automobile crashes. **(T)**	58 (96.7%)	31 (96.9%)	27 (96.4%)	0.92
Item 14. In men, a collar size 17 in. or greater is associated with OSA. **(T)**	41 (68.3%)	18 (56.3%)	23 (82.1%)	0.03
Item 15. OSA is more common in women than in men. **(F)**	41 (68.3%)	19 (59.4%)	22 (78.6%)	0.11
Item 16. CPAP is the first line therapy for severe OSA. **(T)**	48 (80.0%)	24 (75.0%)	24 (85.7%)	0.30
Item 17. Less than 5 apneas or hypopneas per hours is normal in adults. **(T)**	39 (65.0%)	17 (53.1%)	22 (78.6%)	0.04
Item 18. Cardiac arrhythmias may be associated with untreated OSA. **(T)**	57 (95%)	31 (96.9%)	26 (92.9%)	0.48
Total knowledge score out of 18 (mean ± SD)	13.98 ± 2.4	13.19 ± 2.5	14.96 ± 1.7	0.02

* = Chi-square.

The mean total attitude score was 18.0 ± 2.6 out of 25.0 ( Table 3). Senior residents had higher mean total attitude scores compared to junior residents (18.8 ± 1.6 vs. 17.3 ± 3.0, *p* = 0.02). Fifty-nine residents (98.3%) considered OSA as an “important,” “very important,” and “extremely important” clinical disorder. The median values were 4 (IQR 4–5) and 4 (IQR 3–5) for the first and second " importance questions", respectively. The median values were 4 (IQR 3–4), 3 (IQR 3–4), and 3 (IQR 2–4) for the three confidence questions, respectively. Forty-three respondents (71.7%) felt confident in identifying patients at risk for OSA. Twenty-six (43.3%) participants felt confident in their ability to manage OSA. Sixteen participants (26.7%) felt confident in their ability to manage patients on continuous positive airway pressure (CPAP) therapy. The mean score for self-reported confidence in managing patients with OSA was higher in senior residents than in junior residents (3.6 ± 0.9 vs. 3.2 ± 0.9, *p* < 0.01).

**Table 3.** Responses on the attitude segment of the OSAKA questionnaire.

OSAKA Attitude questions	(mean **±** SD)			
	All participants N=60	Juniors N=32	Seniors N=28	*P*-value *
**Importance questions**				
- Importance of OSA as a clinical disorder	4.0 ± 0.8	3.9 ± 0.8	4.2 ± 0.8	0.26
- Importance of identifying patients with possible OSA	4.0 ± 0.7	3.8 ± 0.7	4.3 ± 0.7	0.01
**Confidence questions**				
- Confident in identifying patients at risk of OSA	3.8 ± 0.6	3.7 ± 0.6	4.0 ± 0.6	0.14
- Confident in ability to manage patients with OSA	3.3 ± 0.9	3.2 ± 0.9	3.6 ± 0.9	<0.01
- Confident in ability to manage patients on CPAP therapy	2.9 ± 1.0	2.8 ± 1.0	2.9 ± 1.0	0.59
Total attitude score out of 25 (mean ± SD)	18.0 ± 2.6	17.3 ± 3.0	18.8 ± 1.6	0.02

* = Chi-square.

Age, gender, residency program area, and years of previous ORL practice showed no significant differences in terms of knowledge levels and attitudes toward OSA.

All the included residents were exposed to obese patients, and most of them (98.3%) dealt with patients susceptible to OSA ( Table 4). A total of 81.7% of the respondents had previous exposure to both diagnosed OSA patients and to surgery directed to treat OSA. Only 51.7% of the included trainees had previous exposure to polysomnography data (50% and 53.6% of the junior and senior residents, respectively). For these five clinical exposure elements, senior residents had equal or slightly higher exposure rates than junior residents did. Only 16 participants (26.7%) were aware of sleep disorders (other than sleep apnea).

**Table 4.** Self-reported clinical exposures and awareness of other sleep disorders.

	n (%)				
	All participants N=60	Juniors awareness about other sleep disorders (N=32)		Seniors awareness about other sleep disorders (N=28)	
		Yes	No	Yes	No
**Previous clinical exposure to:**					
- patients susceptible to OSA	59 (98.3%)	31 (96.9%)	1 (3.1%)	28 (100%)	0
- obese patients	60 (100%)	32 (100%)	0	28 (100%)	0
- diagnosed OSA patients	49 (81.7%)	25 (78.1%)	7 (21.9%)	24 (85.7%)	4 (14.3%)
- Polysomnography interpretation	31 (51.7%)	16 (50%)	16 (50%)	15 (53.6%)	13 (46.4%)
- surgery directed to treat OSA	49 (81.7%)	24 (75%)	8 (25%)	25 (89.3%)	3 (10.7%)
**Awareness about sleep disorders** ** (other than sleep apnea)**	16 (26.7%)	5 (15.6%)	27 (84.4%)	11 (39.3%)	17 (60.7%)

Exposure to surgery directed to treat OSA and awareness of sleep disorders (other than sleep apnea) were found to be associated with an increased level of confidence in identifying patients at risk of OSA ( *p* = 0.004 and 0.003, respectively) as well as confidence in the ability to manage patients with OSA ( *p* = 0.01 and 0.02, respectively) ( Table 5).

**Table 5.** Comparison of different clinical exposures to confidence in dealing with OSA patients.

	n (%)						
	All participants N=60	Confidence in identifying patients at risk of OSA (N=60)			Confidence in ability to manage patients with OSA (N=60)		
		Yes N= 43	No N=17	*P*-value *	Yes N= 26	No N=34	*P*-value *
**Previous clinical exposure to:**							
- patients susceptible to OSA	59 (98.3%)	42	17	0.53	26	33	0.38
- obese patients	60 (100%)	43	17	-	26	34	-
- diagnosed OSA patients	49 (81.7%)	34	15	0.41	19	30	0.13
- Polysomnography interpretation	31 (51.7%)	20	11	0.20	11	20	0.21
- surgery directed to treat OSA	49 (81.7%)	39	10	0.004	25	24	0.01
**Aware about sleep disorders** ** (other than sleep apnea)**	16 (26.7%)	16	0	0.003	11	5	0.02

* = Chi-square.

## Discussion

Currently, there is a worldwide increase in the awareness of OSA. This study was designed to evaluate the level of knowledge and attitudes of Saudi ORL trainees toward obstructive sleep apnea.

This study showed that the level of knowledge about OSA among ORL trainee residents included in this study was relatively low compared to their self-reported high level of exposure to patients susceptible to OSA. The mean total knowledge score in our study was 13.98 ± 2.4 out of 18 (equivalent to 77.7%). This was higher than that of most previously published studies that used the same questionnaire. The original OSAKA study had a mean total knowledge score of 13.3 ± 2.8 (73.9%) for their included population (internists, pediatricians, and family practitioners) ^12^. Several other previous studies have used the same tool to assess knowledge in different populations. The mean total knowledge scores among final year medical students in Nigeria, recent medical graduates in Ecuador, general practitioners in Nigeria, internal medicine residents in Nigeria, Latin American primary care physicians, and anesthesiologists in China were 7.6 ± 3.2 (42.2%), 10.4 ± 2.7 (57.5%), 10.0 ± 2.8 (55.6%), 10.8 ± 2.5 (60%), 10.8 (60%), and 11.21 ± 2.89 (62.3%), respectively ^13– 17^. The total knowledge score from using the same questionnaire among Canadian otolaryngology–head and neck surgery (ORL-HNS) residents was reported as the median, which was 16 out of 18 ^18^.

The mean total knowledge score in this study was higher for senior residents than for junior residents, reflecting knowledge gains during residency training. This finding is consistent with the data of Canadian ORL-HNS residents ^18^.

The ORL trainee residents in Saudi Arabia had good recognition of OSA consequences, including hypertension (100%) and a higher incidence of automobile crashes (96.7%). They showed a higher recognition of these two consequences compared with the results of an older study conducted among primary health care physicians in Riyadh, Saudi Arabia ^21^. In our opinion, this difference can be attributed to the current global trend of increased recognition of OSA, training in a specialty dealing with upper airway diseases, and frequent exposure to OSA-susceptible patients.

Positive airway pressure (PAP) therapy is the gold standard first-line treatment for patients with severe OSA ^22^. Question 16 of the knowledge section "CPAP is the first-line therapy for severe OSA" was incorrectly answered by 20% of the participants in our study. This indicates that more education on management schemes is necessary, and more exposure to the available literature on the indications for surgical and non-surgical treatment choices is also required.

Compared to Canadian ORL-HNS residents, the ORL trainee residents in Saudi Arabia had a similar median and a slightly higher IQR level in the two importance questions of the attitudes part in the questionnaire ^18^.

ORL trainee residents in Saudi Arabia had a deficiency in clinical exposure to polysomnography interpretation. In our opinion, their level of exposure to polysomnography should be higher to ensure sufficient experience during their residency training. Our opinion is supported by several facts. First, polysomnography is an important tool for evaluating sleep disorders ^23^. Second, they had a high rate of self-reported exposure to patients who were susceptible to OSA. Third, the SB-ORL curriculum indicated that one of the workshops that trainee residents are recommended to attend is the “Approach to sleep studies” workshop ^19^. One possible reason for this low exposure to sleep studies is the limited number of sleep centers in Saudi Arabia ^24^.

Patients with OSA may also suffer from other comorbid sleep disorders. One good example is insomnia, which is common among patients with OSA ^25^. However, self-reported awareness of sleep disorders other than sleep apnea was low among the ORL trainee residents in Saudi Arabia. This finding can be improved by teaching sessions and mentoring clinics to ensure a comprehensive evaluation and management of patients with OSA.

We found that there is a significant association between self-reported awareness of sleep disorders (other than sleep apnea) and confidence in identifying patients at risk of OSA and confidence in the ability to manage patients at risk of OSA. Again, this association may indicate that more education on comprehensive approaches for OSA patients is required.

Obesity is a known risk factor for OSA ^26^. Obesity is a major health issue worldwide, including in Saudi Arabia ^27^. CPAP is one of the first management choices for adult OSA patients in general and for obese patients with OSA ^22, 28^. Only 26.7% of the included ORL trainee residents in our study felt confident about their ability to manage patients on CPAP therapy. We believe that OSA patients may benefit more from future ORL specialists who have greater capacities to deal with surgical and non-surgical management choices. For this reason, we recommend more education and higher exposure to sleep medicine practices within the ORL specialty or other specialties (such as sleep medicine).

This study has some limitations. First, the response rate in our study was relatively low, yet statistically sufficient. Second, we did not consider the presence or absence of sleep laboratories in the respondents’ hospitals. However, the included subjects mostly followed rotation programs that included secondary and tertiary training hospitals, therefore, minimizing possible related bias. Third, we utilized a popular, validated, and widely used questionnaire (the OSAKA questionnaire). However, this questionnaire mostly includes questions about OSA in adults, with some questions regarding pediatric OSA. These mixed types of questionnaires can be misleading, especially when it comes to confidence in management since the approaches are completely different in these two age groups. We believe that some of these limitations can be avoided in future research.

On the other hand, this study had several strengths. It focused on a single specialty, and it is the first study to be published regarding the knowledge and attitudes toward OSA among ORL trainee residents in Saudi Arabia. In addition, it identified some important and relative clinical exposure variables as well as their correlations with attitudes toward OSA.

## Conclusion

This study describes the current status of OSA knowledge and attitudes among ORL trainee residents in Saudi Arabia. We found that the knowledge levels were acceptable, whereas the attitude scores were lower than expected. Addressing specific studied elements through education and mentored clinical exposure may improve overall training outcomes.

## Data availability

### Underlying data

Figshare: OSA Knowledge and Attitudes. https://doi.org/10.6084/m9.figshare.20339040.v1 ^20^.

This project contains the following underlying data:

-  Raw data.xlsx

### Extended data

Figshare: OSA Knowledge and Attitudes. https://doi.org/10.6084/m9.figshare.20339040.v1 ^20^.

This project contains the following extended data:

-  Data collection form.pdf

-  OSAKA questionnaire.pdf

Data are available under the terms of the Creative Commons Attribution 4.0 International license (CC-BY 4.0)
