# Supplementary material for: Knowledge and attitudes about obstructive sleep apnea among otorhinolaryngology trainee residents in Saudi Arabia: A survey-based cross-sectional study
Source: MedEdPublish (2016). 2022 Aug 17;12:58. [Version 1] doi: 10.12688/mep.19245.1 (PMC10448149; doi:10.12688/mep.19245.1)
Supplement: Peer review history

# Peer review: Reviewer response for version 1

**DOI:** 10.21956/mep.20618.r34436

**Published:** 2023-08-23

**Authors:** Ivana Pavlinac Dodig

**Affiliations:** 1 Neuroscience, University of Split School of Medicine, Split, Croatia

In this survey-based cross-sectional study, the authors aimed to investigate the knowledge and attitudes about obstructive sleep apnea among otorhinolaryngology trainee residents in Saudi Arabia. They revealed that the knowledge levels were acceptable (14 out of 18), whereas the attitude scores were lower than expected. I believe that the topic is important, the findings are interesting, and the manuscript is well-written. To further improve the manuscript, I would suggest that the authors:

1. Report exact numbers (and P values) in the abstract.
2. Provide additional information on OSA in the introduction.
3. Provide information on data distribution normality tests and the reasons why they decided to perform parametric statistics.
4. Report results as numbers (not as words).
5. Table 1, when reporting age remove parentheses.
6. Provide a table showing age, gender, residency program area, and years of previous ORL practice regarding knowledge and attitudes toward OSA as supplementary material.
7. Table 4: omit columns showing “No” data and the last line of the Table (Awareness about sleep disorders). Also, add a column showing P values of comparison between seniors and juniors.
8. Table 5, omit the column showing “All participants”.
9. Provide data on the correlation between knowledge and attitudes.
10. Comment on the paragraph: “Compared to Canadian ORL-HNS residents, the ORL trainee residents in Saudi Arabia had a similar median and a slightly higher IQR level in the two importance questions of the attitudes part in the questionnaire ^18^.”
11. Sentences “Obesity is a known risk factor for OSA ^26^. Obesity is a major health issue worldwide, including in Saudi Arabia ^27^.” should be removed or further elaborated.

Have any limitations of the research been acknowledged?

Yes

Is the work clearly and accurately presented and does it cite the current literature?

Yes

If applicable, is the statistical analysis and its interpretation appropriate?

Yes

Are all the source data underlying the results available to ensure full reproducibility?

Yes

Is the study design appropriate and is the work technically sound?

Yes

Are the conclusions drawn adequately supported by the results?

Yes

Are sufficient details of methods and analysis provided to allow replication by others?

Yes

Reviewer Expertise:

Sleep medicine

I confirm that I have read this submission and believe that I have an appropriate level of expertise to confirm that it is of an acceptable scientific standard.

# Peer review: Reviewer response for version 1

**DOI:** 10.21956/mep.20618.r32532

**Published:** 2022-11-08

**Authors:** Yousef D. Alqurashi

**Affiliations:** 1 Respiratory Care Department, College of Applied Medical Sciences, Imam Abdulrahman Bin Faisal University, Dammam, Saudi Arabia

This study assessed the knowledge and attitudes toward obstructive sleep apnea among otorhinolaryngology trainees in Saudi Arabia. The authors found that the knowledge level about OSA is 14/18, with some deficiency of knowledge about sleep disorders other than OSA. 

 Impression: This is a well-written study and addressed a research gap in Saudi Arabia. I congratulate the authors for conducting such study. 

 Here are few points can strengthen the manuscript:

1. There is no comparison and association data (r2, P value) in the abstract. I think adding such data will catch the readers eyes to continue reading the full article. 
2. The second paragraph in the introduction is good, but is not needed in the introduction. It is enough to mention it in the method section. I suggest to remove it and lengthen the third paragraph by mentioning the studies that assessed the OSA awareness in Saudi Arabia and gulf region.
3. Reporting both the mean and IQR is not recommended. I suggest to remove one of them. 
4. I suggest to add statement like " to the best of our knowledge" when taking about the the uniqueness of this study (second sentence in the last paragraph).

Have any limitations of the research been acknowledged?

Yes

Is the work clearly and accurately presented and does it cite the current literature?

Yes

If applicable, is the statistical analysis and its interpretation appropriate?

Yes

Are all the source data underlying the results available to ensure full reproducibility?

Yes

Is the study design appropriate and is the work technically sound?

Yes

Are the conclusions drawn adequately supported by the results?

Yes

Are sufficient details of methods and analysis provided to allow replication by others?

Yes

Reviewer Expertise:

Sleep medicine

I confirm that I have read this submission and believe that I have an appropriate level of expertise to confirm that it is of an acceptable scientific standard.
